# A Constitutive Relationship between Fatigue Limit and Microstructure in Nanostructured Bainitic Steels

**DOI:** 10.3390/ma9100831

**Published:** 2016-10-14

**Authors:** Inga Mueller, Rosalia Rementeria, Francisca G. Caballero, Matthias Kuntz, Thomas Sourmail, Eberhard Kerscher

**Affiliations:** 1 Department of Mechanical and Process Engineering Materials Testing (AWP), University of Kaiserslautern, Gottlieb-Daimler-Straße, Kaiserslautern 67663, Germany; kerscher@mv.uni-kl.de; 2 Spanish National Center for Metallurgical Research (CENIM-CSIC), Avda. Gregorio del Amo 8, Madrid E-28040, Spain; rosalia.rementeria@cenim.csic.es (R.R.); fgc@cenim.csic.es (F.G.C.); 3 Robert-Bosch GmbH, Materials and Process Engineering Metals, Renningen, Stuttgart 70465, Germany; matthias.kuntz2@de.bosch.com; 4 Asco Industries CREAS, Avenue de France, BP 70045, Hagondange Cedex 57301, France; thomas.sourmail@ascometal.com

**Keywords:** fatigue limit, crack growth, nanostructured bainite, crystallography, EBSD, critical crack length, Kitagawa diagram

## Abstract

The recently developed nanobainitic steels show high strength as well as high ductility. Although this combination seems to be promising for fatigue design, fatigue properties of nanostructured bainitic steels are often surprisingly low. To improve the fatigue behavior, an understanding of the correlation between the nanobainitic microstructure and the fatigue limit is fundamental. Therefore, our hypothesis to predict the fatigue limit was that the main function of the microstructure is not necessarily totally avoiding the initiation of a fatigue crack, but the microstructure has to increase the ability to decelerate or to stop a growing fatigue crack. Thus, the key to understanding the fatigue behavior of nanostructured bainite is to understand the role of the microstructural features that could act as barriers for growing fatigue cracks. To prove this hypothesis, we carried out fatigue tests, crack growth experiments, and correlated these results to the size of microstructural features gained from microstructural analysis by light optical microscope and EBSD-measurements. Finally, we were able to identify microstructural features that influence the fatigue crack growth and the fatigue limit of nanostructured bainitic steels.

## 1. Introduction

Nanostructured bainite is obtained by isothermal transformation after austenitization of high-carbon, high-silicon steels. The structure after transformation roughly consists of carbon-enriched retained austenite dispersed in a matrix of nanometer-scale plates of bainitic ferrite [1]. The static properties of these steels reach interesting levels, with ultimate tensile strengths over 2 GPa in combination with 20% total elongation [2,3]. The material is commercially available as armors and shafts [4], and several research groups are carrying out work to assess the in-use properties and further spread the applications [3,5,6,7,8,9].

For fatigue design, a high ultimate tensile strength as well as a high ductility are desirable. Nanobainitic steels show both, but, nevertheless, their fatigue limit is often surprisingly low [2]. The reason for this is not totally understood yet.

**Figure 1:**
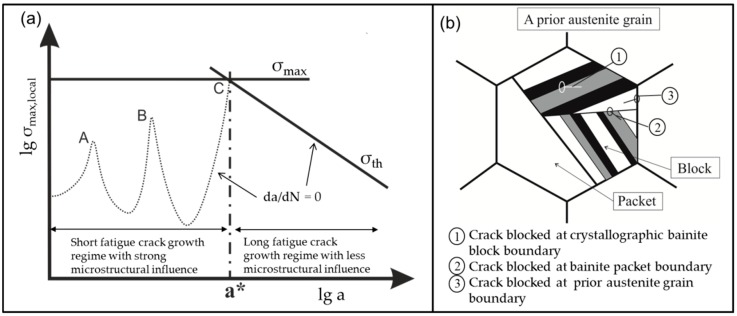
(**a**) Scheme of Kitagawa diagram according to [10]; and (**b**) scheme of nanobainitic microstructure according to [11] with possible barriers for a growing fatigue crack.

Enhancing the fatigue limit of high strength steels means improving the ability of the microstructure to decelerate a growing fatigue crack. Totally avoiding fatigue cracks and their initiation is not possible and not necessary because there are always fatigue cracks existing in the microstructure as shown by Miller [10]. At the same time, Miller discussed that the microstructure has a strong influence on growing fatigue cracks, and, as a consequence, also on the fatigue limit. This correlation is shown in the Kitagawa-diagram [10], in which the borders of crack growth are given by lines (dotted line and straight lines named *σ*_th_) representing a crack velocity *da*/*dN* = 0 (see Figure 1a). In the first part of the diagram the fatigue crack growth is strongly influenced by microstructural features, such as twin boundaries, grain boundaries, and phase boundaries, -represented in Figure 1a as peaks A, B, and C, respectively, of the dotted line. These features act as barriers for a growing crack because the crack needs a higher driving force to overcome those barriers. If cracks are not able to cross those barriers, the material will not fail. To predict the highest possible stress range at which the cracks are still not able to grow across those barriers, a crucial understanding of the microstructure is necessary to identify the main barrier in the microstructure. In principle, several barriers can exist in the microstructure until the crack reaches the major barrier, marked as C in Figure 1a. After overcoming this major barrier, a fatigue crack will continue to grow until final failure. In this point C, the critical crack length a* is reached. If the stress amplitude is high enough to let the crack grow longer than the critical crack length, the crack cannot be stopped anymore by microstructural features and will lead to failure of the specimen.

Microstructural barriers in bainitic steels are a result of the displacive transformation of the austenite. In displacive transformations, the close-packed {111} plane of the austenite (face-centered cubic, fcc) is parallel to a {110} plane of the product phase (body-centered cubic, bcc or body-centered tetragonal, bct). In nanobainitic steels, most of the bainitic plates and the parent austenite interfaces are found to have an orientation relationship (OR) close to the Nishiyama–Wassermann (N–W), {111}γ||{110}α with <110>γ||<001>α [12,13]. A crystallographic packet is defined as a group of crystallographic variants with a common {111} austenite plane. Each bainitic packet can be divided into bainitic blocks of the three variants of the N–W relationship satisfying the same parallel plane relationship. In consequence, the microstructure obtained is highly misoriented featuring various potential microstructural barriers. In Figure 1b, a scheme of the nanobainitic microstructure is given according to [11] with possible barrier for a growing fatigue crack. These could be the boundaries of prior austenite grains, packets or bainitic blocks.

In previous work, a microstructural examination at the Stage I of fatigue crack propagation showed that a growing crack deflects at the interphase boundaries between blocks and packets of the ferritic phase, but not in the interphase boundaries within a single block [14]. This suggests that the bainite block size is the crystallographic parameter controlling the crack propagation at this stage [14].

The aim of the present paper is to identify the microstructural features that do influence the fatigue crack growth and ultimately the fatigue properties of nanostructured bainitic steels. With this purpose, we have performed fatigue tests, crack growth experiments, and correlated these results to the size of microstructural features gained from microstructural analysis and EBSD-measurements.

## 2. Materials and Methods

### 2.1. Materials

The chemical compositions of the investigated alloys are 0.6C−(1.5 and 2.5)Si−1.3Mn−1.7Cr and 1.0C−(1.5 and 2.5)Si−0.7Mn−1.0Cr, wt %. Two of the materials (1C-1.5Si-950-250-16 and 0.6C-2.5Si-890-250-16) are laboratory casts, specially manufactured for these investigations. The other four are commercial industrial casts (see Table 1). Specimens were austenitized for 1 h and subsequently isothermally austempered after quenching, as listed in Table 1. Specimens have been encoded according to the material and heat treatment, expressed as (*x*C-*y*Si)-*T_γ_*-*T_b_*-*t_b_*, where *x* and *y* are the C and Si contents of the steel (wt %), *T_γ_* and *T_b_* are the austenitization and austempering temperature, respectively, and *t_b_* is the bainite transformation time (see Table 1).

### 2.2. Micorstructural Characterization

The microstructural characterization was carried out with different techniques to analyze the microstructure at different levels.

To assess the correlation between the fatigue limit and the bainite block size, an efficient methodology must be developed to evaluate the latter in an efficient, systematic and statistically representative way. Electron backscatter diffraction (EBSD) mapping of large areas (containing several prior austenite grains) cannot be performed in a reasonable time, since a step size of tenths of microns must be maintained for successful indexing. In this sense, color-contrast in optical micrographs is known to correlate with crystal orientations, different phases or boundary regions [15]. To assess the correspondence between the crystallographic features observed by EBSD and the contrast observed in optical micrographs, EBSD analyses were first performed in selected areas of the 1C-2.5Si-950-250-16 and 0.6C-1.5Si-890-250-16 samples. In a second step, samples were Nital-etched and the areas mapped by EBSD were observed by light optical microscopy (LOM). Detailed information regarding the different investigation methods are given in the following.

#### 2.2.1. Specimen Preparation

Samples for LOM observations and EBSD investigation were cut in longitudinal cross sections from the head shoulder of fatigued specimens. The samples where ground and polished by means of a diamond paste with a diamond grain size of 1 μm. Samples for LOM observation intended for the microstructural analysis to determine microstructural sizes were then etched with 3% Nital.

Samples for EBSD investigation intended for the comparison of LOM and EBSD were subjected to several cycles of etching and polishing to obtain an undeformed surface followed by 50 nm colloidal silica finishing. After the EBSD measurement, these samples were prepared for the comparative LOM observation. They were etched with 2% Nital in several steps alternating with carefully polishing to remove the passive layer generated by the EBSD electron beam and to receive the visible microstructure in the same area as the EBSD measurement was carried out. Additionally indentation marks, inserted before EBSD-measurement helped to localize the area of EBDS analysis for LOM observation of the same area.

#### 2.2.2. Light Optical Microscopy (LOM)

For the comparison of LOM and EBSD investigation, optical micrographs were obtained by means of a Nikon Epiphot 200 light optical microscope (Nikon, Otawara, Japan) in the same area as the EBSD measurement was carried out.

**Figure 2:**
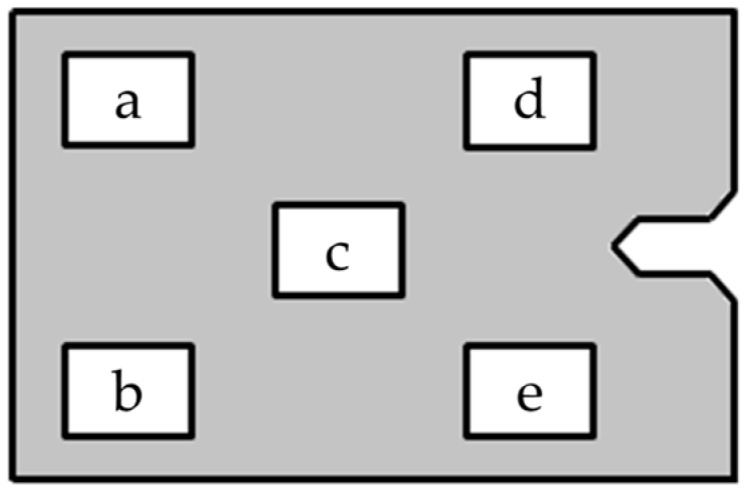
Scheme of the longitudinal section of the fatigue specimens and the five positions (**a**–**e**) where LOM observations were performed.

For further microstructural analysis, optical micrographs were obtained by means of a Leica DM 2500 M light optical microscope (Leica, Wetzlar, Germany) in five different areas, each of a size of 117.5 mm^2^, of each specimen to get a representative inspection area of 587.5 mm^2^ in total, as illustrated in Figure 2.

#### 2.2.3. Electron Backscatter Diffraction (EBSD)

EBSD was undertaken on a Zeiss Auriga Compact FIB-SEM (Zeiss, Göttingen, Germany) operating at 15 kV and fitted with an Oxford NordlysNano EBSD detector (Oxford-Instruments, Oxford, UK) interfacing with the AZtec software suite (AZtec, Oxford, UK) A step size of 0.075 μm was maintained over an area of about 43 × 57 μm^2^.

#### 2.2.4. Field Emission Gun-Scanning Electron Microscopy (FEG-SEM)

Specimens for FEG-SEM observations where cut in cross sections from the grips of tensile test samples. Sample preparation was performed following the standard metallographic procedures with 1 μm diamond paste finishing. A 2% Nital etching solution was used to reveal the microstructure going over several cycles of etching and polishing to favor the etchant penetration. FEG-SEM examination was performed on a Hitachi S-4800 gun scanning electron microscope (Hitachi, Tokyo, Japan) operating at 7 kV.

### 2.3. Fatigue Tests

**Figure 3:**
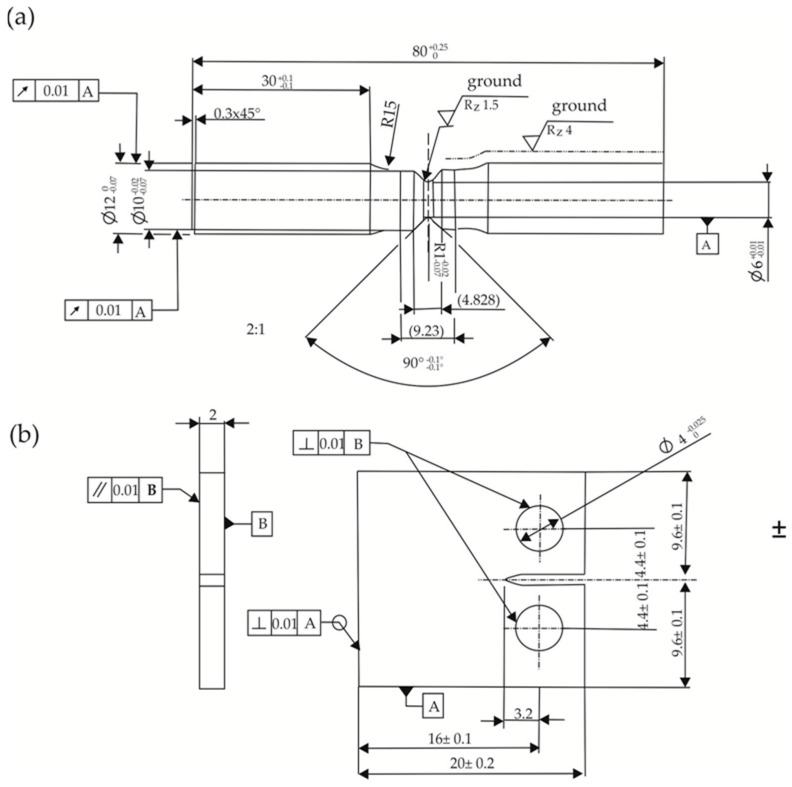
(**a**) Draft of round specimen with *K_t_* = 2 for fatigue testing; and (**b**) draft of CT-Specimen for crack growth experiments.

Fatigue tests were carried out stress-controlled on a resonance pulser with a frequency of 150 Hz with sine wave load. Tests were carried out for all materials’ conditions with notched round specimens with a notch factor of *K_t_* = 2. The notch factor describes the ratio between the maximum principle stress in the cross section of a notched specimen and the nominal stress in an unnotched specimen. For some conditions, additional tests were done on specimens with a notch factor of *K_t_* = 4. A draft of these specimens is shown in Figure 3a. All of these tests were done with a load ratio, i.e., the ratio of the minimum stress and the maximum stress of the sine wave during fatigue tests, of *R* = 0.1. Additionally, fatigue tests were done on notched specimens with *K_t_* = 2 and a load ratio of *R* = −1. For further information regarding the combination of test parameters, see Table 2. Woehler-curves were generated by fatigue experiments according to the stair-case-method with at least 15 specimens of each variant, and the fatigue limit was determined out of the results with the probit-method.

The fracture surfaces of the broken specimens were investigated by scanning electron microscopy (SEM) by means of a Philips XL40 (Philips, Amsterdam, The Netherlands).

### 2.4. Crack Growth Experiments

CT-specimens for crack growth experiments were designed according to the standard ASTM E647 [16] with a smaller scale than suggested because of the limited size of the available material. The draft of these specimens is shown in Figure 3b. The notch was machined by wire cutting and the upper and lower surfaces of the specimens were ground to guarantee the parallelism after heat treatment. These tests were done with all materials and heat treatment conditions. For each condition, two specimens were tested. Before testing the upper and lower surfaces, the specimens were polished with diamond paste down to 3 µm.

Crack growth experiments were carried out stress-controlled on a servo hydraulic test stand with a load ratio of *R* = 0.1 according to the standard ASTM E647. During loading with a sine wave the crack growth was observed by a microscope camera. The digital microscope camera used for the tests was an Inspectra from Reflecta (Reflecta, Eutigen, Germany) with two possible magnifications of 50× and 200×. The software used to record the growing fatigue crack was Digital Viewer II from Reflecta (Reflecta, Eutingen, Germany).

The initial crack was inserted with a maximum force *F_max_* of 1200 N under cyclic loading also with a load ratio of *R* = 0.1 until the crack reached a length of about 300–500 µm. The force *F_max_* corresponds to a *K_I,max_* of about 20 MPam^1/2^ in the notch ground before crack initiation. *K_I,max_* was calculated with the following equation according to the standard ASTM E647:

(1) $$K_{I,max}=\frac{F_{max}}{\left(B\cdot W^{1/2}\right)}\cdot \frac{\left(2+\alpha \right)}{{\left(1-\alpha \right)}^{\frac{3}{2}}}\cdot \left(0.886+4.6\alpha -13.32\alpha^{2}+14.72\alpha^{3}-5.56\alpha^{4}\right),$$

where *B* is the thickness of the specimen, *W* is the distance from the center of the drill hole to the end of the specimen, *α* is the ratio of total crack length a and thickness *B* of the specimen. As a starting crack length, *a* = 3.2 mm was assumed (compare Figure 3b).

The length of the initial crack was measured by SEM by means of a Philips XL40. The following crack growth experiments were done stepwise with decreasing *K_I,max_* to investigate threshold values for long crack growth *K*_th_. A step was either defined by a visible crack elongation in the microscope camera, which was fixed at the servo hydraulic test stand, for high *K_I,max_* or for lower *K_I,max_* with a very low crack elongation that could not be detected by microscope camera a step was defined by 100,000 cycles. After each step, the specimens were observed by means of SEM and the crack elongation Δa was measured. With help of the new crack length, a new, lower *K_I,max_* was calculated, i.e., a lower force *F_max_* was determined for the next step. This procedure was repeated until a load was reached at which no crack growth could be detected by SEM.

During every step, the load cycles were counted to determine the medium velocity of the crack per cycle *da*/*dN*. To receive crack growth curves from the individual results of each step, all data points corresponding to the same material and heat treatment condition were fitted to the Erdogan–Ratwani equation [17]

(2) $$\frac{da}{dN}=C_{E}\frac{{\left(K_{I,C}-K_{I,max}\right)}^{m_{E}}}{\left(1-R\right)K_{I,C}-K_{I,max}}$$

with load ratio *R* = 0.1, fracture toughness *K_I,C_* fixed at 23 MPa × m^1/2^, threshold value for long crack growth *K*_th_ taken from crack growth results, empirical constants *C_E_* and *m_E_* fitted with the software Origin lab (Origin 2015G, Origin Lab Corporation, Northampton, MA, USA). Threshold values *K*_th_ were determined from the fitted curves at a crack velocity *da*/*dN* of 10^−7^ mm/cycle.

## 3. Results

### 3.1. General Microstructural Examination

**Figure 4:**
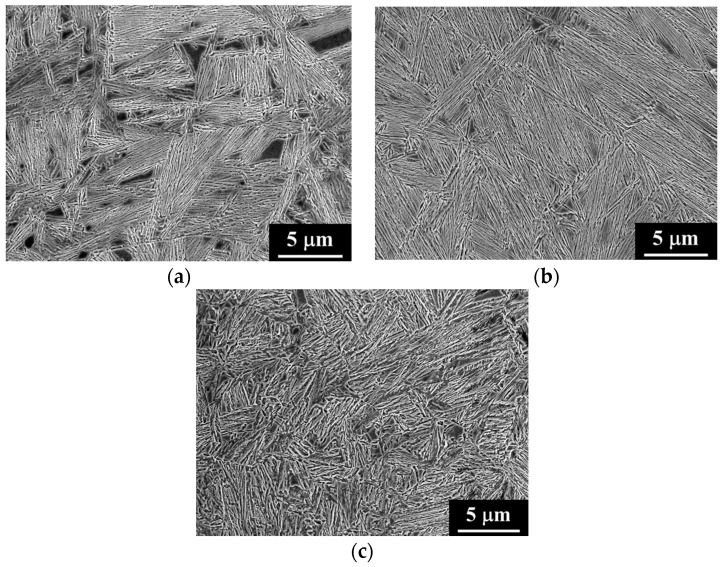
FEG-SEM micrographs for (**a**) 1C-2.5Si-950-250-16; (**b**) 1C-2.5Si-950-250-40; and (**c**) 0.6C-1.5Si-890-250-16 specimens.

The morphology of the microstructure was observed by means of FEG-SEM on etched surfaces. The structures obtained consist mostly of nano-scale bainitic ferrite plates and dispersed regions of retained austenite, as illustrated in Figure 4. Retained austenite features appear in relief with respect to bainitic ferrite on the etched surface exhibiting two morphologies: thin films between the bainitic ferrite plates and blocks between sheaves of bainite (groups of parallel bainitic ferrite plates).

Figure 4a,b show the structures for the 1C-2.5Si steel transformed at 250 °C. It is evident that retained austenite blocks are present in greater proportion for the shorter heat treatment. In the case of the 0.6C-1.5Si-890-250-16 material, the micrograph Figure 4c shows little presence of retained austenite blocks and a smaller length of the bainite sheaves.

#### EBSD Examination

**Figure 5:**
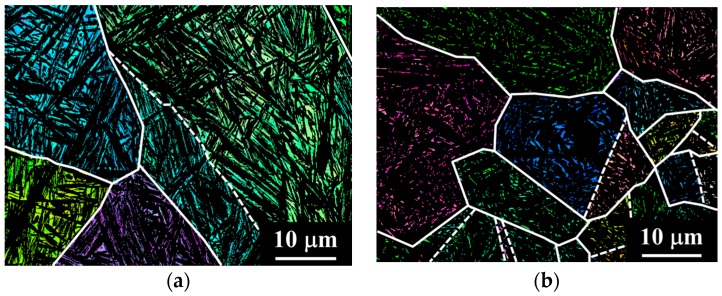

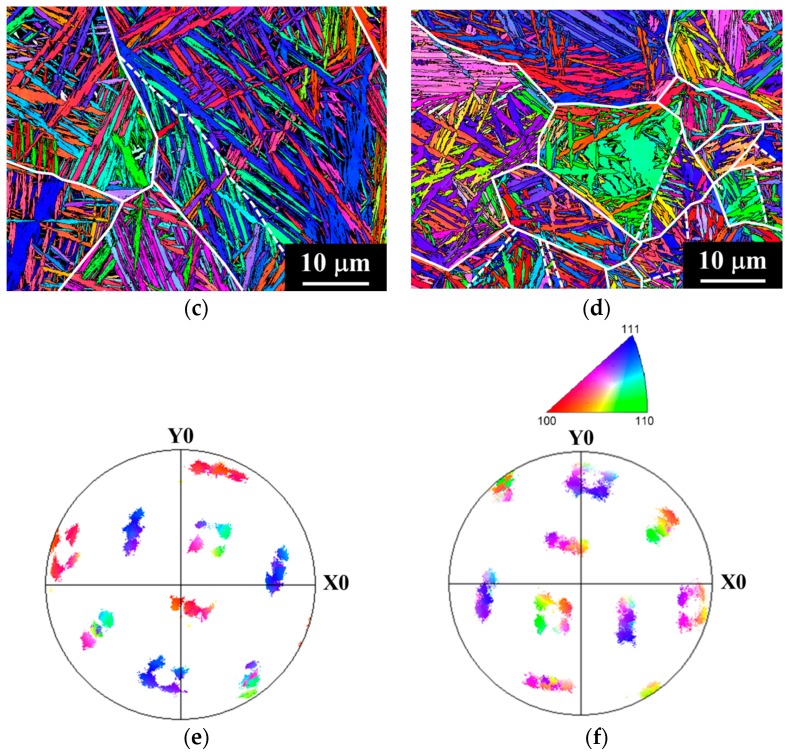
EBSD Inverse Pole Figure (IPF) of the austenite phase in (**a**) 1C-2.5Si-950-250-16 and (**b**) 0.6C-1.5Si-950-250-16 specimens; corresponding EBSD Inverse Pole Figure of the ferrite phase in (**c**) 1C-2.5Si-950-250-16 and (**d**) 0.6C-1.5Si-950-250-16 specimens; EBSD Pole Figure for the ferrite phase formed from a single prior austenite grain in (**e**) 1C-2.5Si-950-250-16 and (**f**) 0.6C-1.5Si-950-250-16 specimens. Prior austenite grains boundaries are delimited by solid white lines whereas prior austenite twin boundaries are delimited by dashed white lines.

Figure 5a,b show the Inverse Pole Figure (IPF) color maps of the austenite in the 1C-2.5Si-950-250-15 and 0.6C-1.5Si-950-250-16 specimens, respectively. The corresponding IPF maps of the ferrite are shown in Figure 5c,d for the 1C-2.5Si-950-250-16 and 0.6C-1.5Si-950-250-16 samples, respectively. The colors correspond to the crystallographic orientation normal to the observed plane, each color representing a different crystallographic orientation. The black boundaries were drawn where the misorientation angle is greater than 10°, or the other phase is present. Prior austenite grain boundaries are drawn with white-solid lines, whereas twin boundaries are drawn in white-dashed lines in both austenite and ferrite IPF maps. Figure 5e,f show the show the correspomnding pole figure (PF) of a single prior austenite grain in Figure 5c,d respectively, where blocks have an OR close to N-W [18]. It is revealed that a prior austenite grain was devided by bainitic packets consisting of three bainitic blocks where each block contains a single variant of the bainitic lath, as previously reported [12,14].

**Figure 6:**
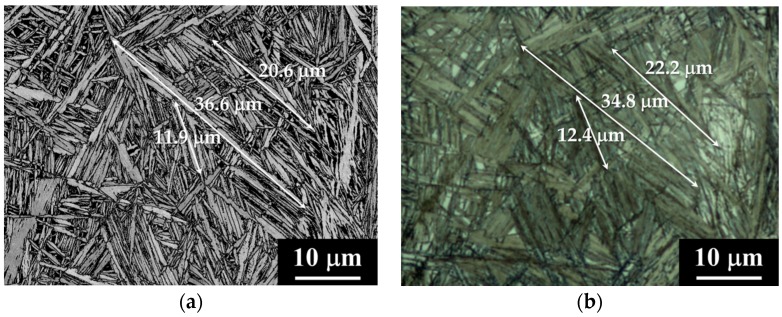
EBSD-LOM correspondence for the 1C-2.5Si-950-250-16 specimen (**a**) EBSD Band Contrast map for the ferrite phase where black lines represent the crystallographic block boundaries; and (**b**) corresponding light optical micrograph. The **white** arrows indicate the length of the crystallographic blocks as measured with both techniques.

Figure 6a shows the block boundaries in black lines superimposed to the Band Contrast (BC) map in the 1C-2.5Si-950-250-16 sample and Figure 6b shows the corresponding optical micrograph. Though some of the crystallographic blocks observed by EBSD appear blurry by LOM, the correspondence is clear the other way, i.e., features that can be undoubtedly distinguished by LOM correspond to crystallographic blocks as observed by EBSD. The arrows in Figure 6a,b indicate the length of some of these crystallographic blocks, showing that measurements performed with both techniques are in good agreement. The sizes of the features observed by LOM and EBSD being comparable, it can be said, that features detected and measured using LOM are crystallographic bainite blocks. This fact proves that we are able to determine crystallographic sizes like the sizes of crystallographic bainite blocks directly from micrographs observed by LOM. This method is timesaving and the inspected area in LOM is many times larger than in EBSD. For further investigations, an average of the three largest measured length *l*_block_ and width *c*_block_ of bainite blocks at all five positions (see Figure 2) was calculated from the light optical micrographs. The largest dimensions were used because they are most harmful for fatigue crack growth. Results of the measured sizes are shown in Table 2.

### 3.2. Fatigue Tests

**Figure 7:**
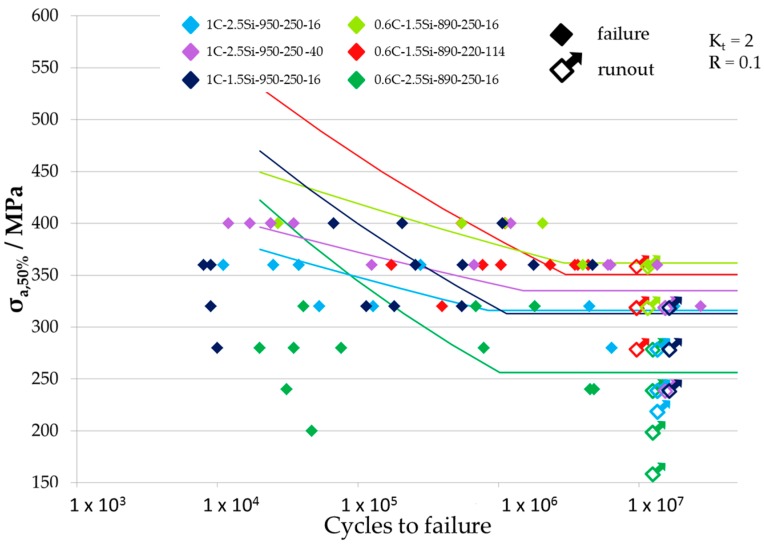
Woehler curves of the variants tested at specimens with *K_t_* = 2 and a load ratio *R* = 0.1.

**Figure 8:**
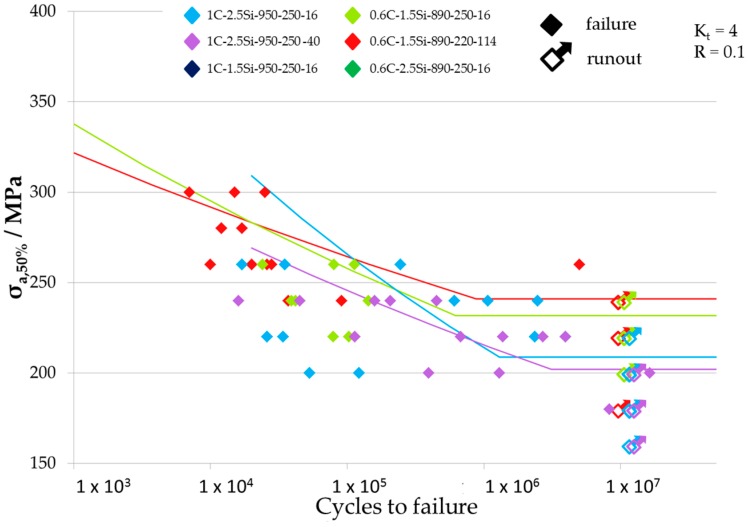
Woehler curves of the variants tested at specimens with *K_t_* = 4 and a load ratio *R* = 0.1.

**Figure 9:**
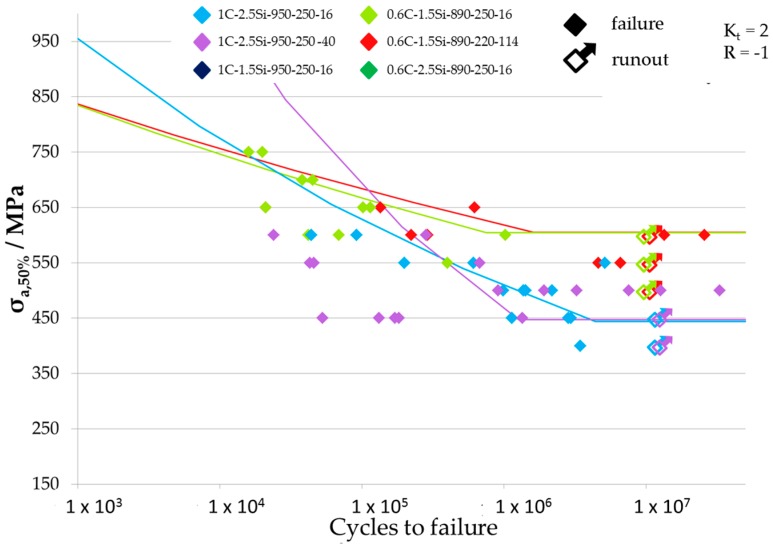
Woehler curves of the variants tested at specimens with *K_t_* = 2 and a load ratio *R* = −1.

Results of fatigue tests are given in Table 2 where all fatigue limits for the different tested load ratios and notch geometries are listed. The resulting Woehler-curves are shown in Figure 7, Figure 8 and Figure 9 for the different tested notch geometries and load ratios. Figure 7 shows the Woehler-curves for *K_t_* = 2 and *R* = 0.1, Figure 8 shows Woehler-curves for *K_t_* = 4 and *R* = 0.1, and the Woehler-curves for *K_t_* = 2 and *R* = −1 are shown in Figure 9. Results show a large scatter in lifetime, which is usual for high strength steels. For all testing parameters, 0.6C-1.5Si microstructures exceeded the fatigue limits of all material conditions with a carbon content of 1 wt %.

**Figure 10:**
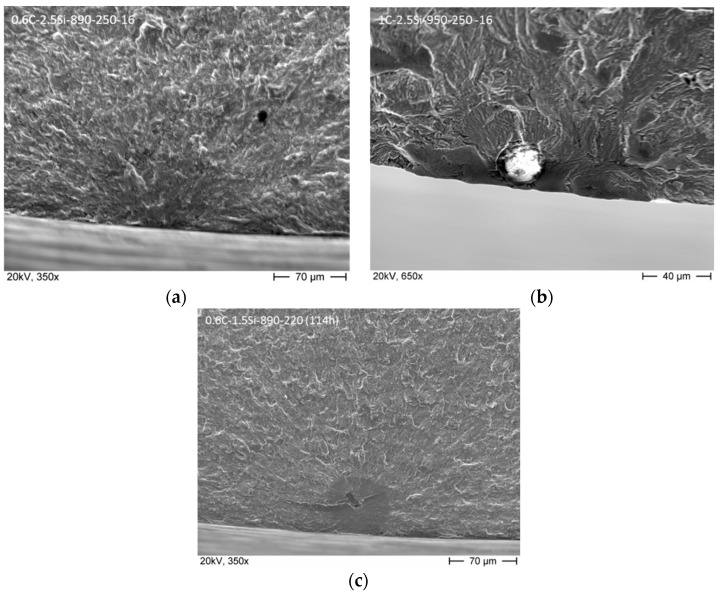
Results of fracture surface analysis. (**a**) Crack initiation at the surface; (**b**) crack initiation at Al_2_O_3_ inclusion near the surface; and (**c**) crack initiation at TiN subsurface inclusion.

A fracture analysis of all broken specimens was carried out. The fracture surfaces of all broken specimens, and, especially, the crack initiation point was investigated by SEM. It was found that a crack could initiate either at the surface or at nonmetallic inclusions located at the surface or in the interior of the material. Two different types of inclusions were determined: Al_2_O_3_- and TiN-inclusions. These different crack initiation points led to the large scatter within the obtained results. Detailed results are published elsewhere [19]. Figure 10 shows examples of the different crack initiation points. In Figure 10a, an example for crack initiation at the surface of the specimen is shown. Figure 10b shows an Al_2_O_3_-inclusion near the surface serving as crack initiation and Figure 10c a TiN-inclusion in the matrix material as crack initiation point.

During the fracture analysis, macro cracks were found on the fracture surfaces of the two laboratory casts (1C-1.5Si-950-250-16 and 0.6C-2.5Si-890-250-16). These macro cracks were already in the material before fatigue tests. In some specimens, these macro cracks also led to crack initiation and thereby falsified further interpretation.

### 3.3. Crack Growth Experiments

**Figure 11:**
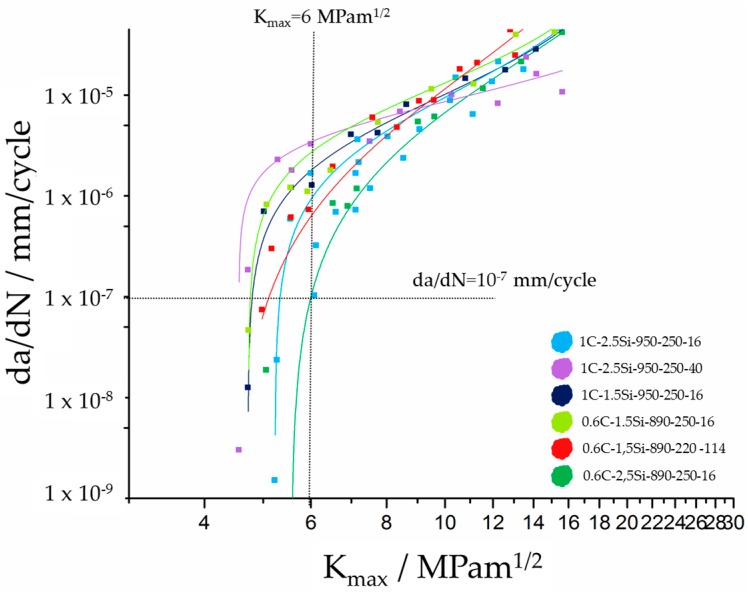
Crack growth curves resulting from crack growth experiments at CT-specimen [19].

Figure 11 shows the crack growth curves fitted according to Erdogan-Ratwani [17]. Table 2 gives the values of threshold for long crack growth *K*_th_ determined at a crack velocity of 10^7^ mm/cycle.

The fitted crack curves are well determined and show all approximately the same crack growth behavior except of one curve. The purple one corresponding to 1C-2.5Si-590-250-40 is crossing all other crack growth curves in a manner that suggests a different crack growth behavior especially in the Paris regime. In contrast, the threshold values for long crack growth *K*_th_ are pretty much alike and vary only between 4.6 and 5.3 MPam^1/2^ except for 0.6C-2.5Si-890-250-16, which exceeds the other values with a threshold value for long crack growth *K*_th_ of 6 MPam^1/2^.

## 4. Discussion

Improving the fatigue behavior means understanding the role of the microstructure regarding fatigue crack growth. In general, the fatigue crack growth can be classified into two regimes: the regime of short fatigue crack growth and the regime of long fatigue crack growth. In the regime of short fatigue crack growth, the microstructure has a strong influence on the crack velocity and the crack path [10]. A crack is defined as short until it reaches the critical crack length a* as marked in the Kitagawa diagram in Figure 1a. Then, the crack enters the long crack regime with only a small microstructural influence that vanishes more and more with increasing crack length. To get a fundamental understanding regarding the fatigue behavior of nanostructured bainitic steels, we need to learn about the microstructural features playing a role in the fatigue crack growth and crack stop.

We used broken fatigue specimens to learn about the conditions enabling crack growth, as it is not possible to get microstructural information regarding the crack growth behavior from runouts because it is hard or impossible to find cracks in run-out specimens. The fracture analysis of all broken specimens showed different crack initiation points: the surface of the specimen, nonmetallic inclusions located at the surface, and subsurface inclusions. To compare those to the sizes of the measured crystallographic bainite blocks, stress intensity factors (SIF) for failure at nonmetallic inclusions were calculated according to Murakami [20,21]. He distinguishes two cases with *A* = 0.65 for an inclusion at the surface and *A* = 0.5 for a subsurface inclusion in the following formula:

(3) $$K_{incl}=A\cdot \sigma_{max,local}{\left(\pi \cdot area_{incl}^{\frac{1}{2}}\right)}^{\frac{1}{2}}.$$

The maximum local stress of failure at the crack initiation point, which was at a surface or at a subsurface position, was used for calculation taking into account the stress distribution in the notched area. Additionally, this calculated local stress was reduced by its elastic part with the help of Neuber’s rule [22] because we supposed that only the remaining plastic part of the maximum local stress *σ*_max,local_ plays a role for crack initiation and crack growth.

In addition, stress intensity factors at crystallographic bainite blocks were calculated for all specimens. Due to their shape, crystallographic bainite blocks are treated as very shallow cracks orientated horizontally to the surface of the specimen according to [20,21]. For calculation, the following equations were used, depending on the ratio of *l*_block_ and *c*_block_:

(4) $$K_{block}=0.65\cdot \sigma_{max,local}{\left(\pi \cdot area_{block}^{1/2}\right)}^{1/2},$$

(5) $$\mathrm{with}\;area_{block}^{1/2}={\left(l_{block}\cdot c_{block}\right)}^{1/2}\;for\;l_{block}/c_{block}<10,$$

(6) $$\mathrm{with}\;area_{block}^{1/2}=10^{1/2}\cdot c_{block}\;for\;l_{block}/c_{block}\geq 10.$$

**Figure 12:**
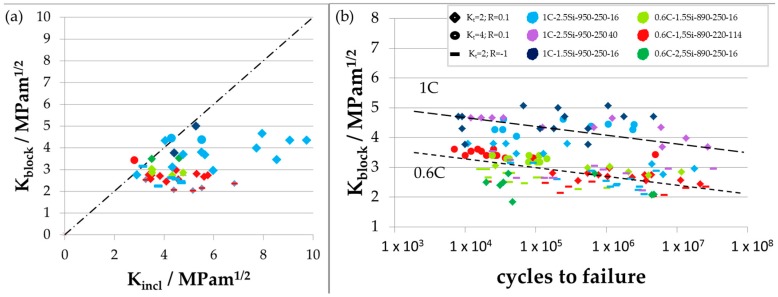
(**a**) SIF at nonmetallic inclusions plotted against SIF at crystallographic bainite blocks; and (**b**) SIF at crystallographic bainite blocks plotted against cycles to failure. The two dotted lines represent two groups of SIF with a linear decreasing trend, which are formed in the diagram.

In Figure 12a, these values are compared to each other. The SIF at nonmetallic inclusions is higher than or equal to the SIF at crystallographic bainite blocks. This means that SIF at nonmetallic inclusions are more critical for fatigue crack initiation than SIF at crystallographic bainite blocks and failure is expected to occur from nonmetallic inclusions as observed. The influence of detrimental flaws like nonmetallic inclusions on the fatigue limit is already well described in literature [20,21,23,24]. Because of the overlaying influence of detrimental flaws on the fatigue limit, it is hard to analyze only the effect of microstructural features on the fatigue limit. Therefore, we analyzed the correlation between microstructural sizes like bainite block size and the fatigue behavior. In Figure 12b, SIFs at crystallographic bainite blocks are plotted versus the corresponding cycles to failure showing a decreasing slope for all states. Here, two groups are visible. The upper group corresponds to the two variants 1C-2.5Si-950-250-40, for which fatigue limits were determined with a slightly different notch geometry of *K_t_* = 1.89, and 1C-1.5Si-950-250-16, which is one of the two laboratory casts in which macro cracks were found, which lead to fatigue failure in some cases. The other variants are forming the lower group, but the 1C-2.5Si-950-250-16 variant is scattering between both.

Results for the bainite crystallographic block length in Table 2 indicate that there is a correlation between the maximum block length and the austenitization temperature, i.e., between the maximum block length and the prior austenite grain size. Higher austenitization temperatures lead to larger prior austenite grain sizes and these lead to longer crystallographic blocks, as Figure 6 confirms. On the other hand, prior austenite grains show thermal twinning prior to bainitic deformation, as revealed in Figure 6a,b. The presence of twins limits the maximum length of bainite blocks within a single prior austenite grain to the length of the *effective austenite grains*, i.e., to the regions where the austenite has approximately a unique orientation with an inner misorientation below 10° [25]. The IPF maps shown in Figure 6c,d reveal that the maximum length of a bainite block is limited by the size of the effective austenite grain size. In these structures, there is usually one bainite block crossing the effective austenite grain from one side to another, which would further limit the length of the rest of the blocks by impingement. As a consequence, crystallographic blocks in structures produced from 0.6C steels austenitized at 890 °C are shorter than those produced from 1C steels austenitized at 950 °C.

The correlation between austenitizing temperature and the maximum block length cannot be the reason for the partitioning into two groups in Figure 12b. As the two austenitizing temperatures are mixed up in the lower group, this separation is not an effect of the austenitizing temperature and the resultant difference in prior austenite grain size. However, the two groups form a threshold value, which gives a first hint that the crystallographic bainite blocks govern the fatigue behavior.

The decreasing lines of the SIFs at crystallographic bainite blocks show that these blocks have an influence on the fatigue behavior. Because of that and because of results of further investigations [14], we assume that a fatigue crack running across a crystallographic bainite block can be stopped at the strongest barrier within the nanobainitic structure if the local stress is lower than the fatigue limit, which means also that the local stress intensity factor is lower than *K*_th_.

**Figure 13:**
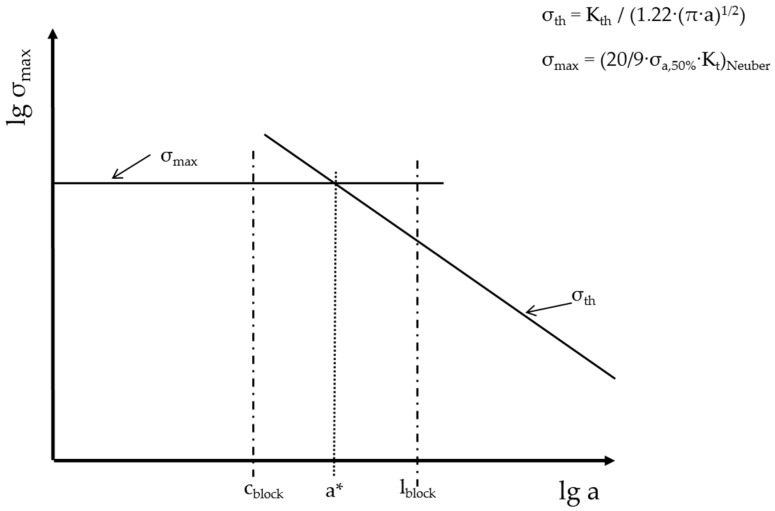
Scheme of Kitagawa diagram drawn with results gathered from crack growth experiments and fatigue tests.

With the gathered results from the different tests carried out, it is possible to draw Kitagawa diagrams for all material states and testing conditions in the way shown in Figure 13. Due to the lack of exact information regarding the microstructural influence in the short crack regime so far, we assume an upper limit *σ*_max_ for fatigue crack growth calculated from the determined fatigue limits *σ*_a,50%_ taking into account the geometry of the notch and reducing the elastic stress in the notch ground according to Neuber [22]. For the limit *σ*_th_ in the right decreasing part of the diagram, we use the threshold value for long crack growth *K*_th_, which was determined with crack growth experiments. This leads to the decreasing small dashed line *σ*_th_.

From these diagrams, we receive different information. In the first place, we can determine the critical crack length a*, which is the intersection point between *σ*_max_ and *σ*_th_. After reaching this barrier, a growing fatigue crack changes from short to long crack growth regime. In the latter case, the microstructure loses its influence on the fatigue crack growth and a fatigue crack is unstoppable under constant amplitude loading. Results of the critical crack length a* are given in Table 2 for the different notch geometries and load ratios during testing. Compared to already determined microstructural sizes, the critical crack length a* is in the order of magnitude of the size of the bainite block width *c*_block_. More precisely, the size of the critical crack length a* lies between the size of the crystallographic bainite block width *c*_block_ and the block length *l*_block_. Two cases are conceivable: a crystallographic bainite block width *c*_block_ that is smaller than the critical crack length a* and one larger than the critical crack length a*. The first case leads to bainite block boundaries that can act as barriers for a growing short fatigue crack because a fatigue crack reaches a block boundary before its length is equal to the critical crack length a*. This applies to the three 0.6C conditions and 1C-2.5Si-950-250-16, which are the materials and heat treatments forming the lower decreasing group in Figure 12b. In the second case, a fatigue crack reaches its critical crack length before reaching a crystallographic bainite block boundary and the bainite block boundary cannot act as barrier for a growing crack. This applies for the two material states 1C-1.5Si-950-250-16 and 1C-2.5Si-950-250-40, which are laying in the upper decreasing group in Figure 12b. However, in the last two cases, we need to question the interpretation because the first variant (1C-1.5Si-950-250-16) is one of the laboratory casts with macro cracks and the second variant (1C-2.5Si-950-250-40) was tested under slightly different conditions with a slightly lower notch factor, as already mentioned. Both can influence the results and thereby also the interpretation.

**Figure 14:**
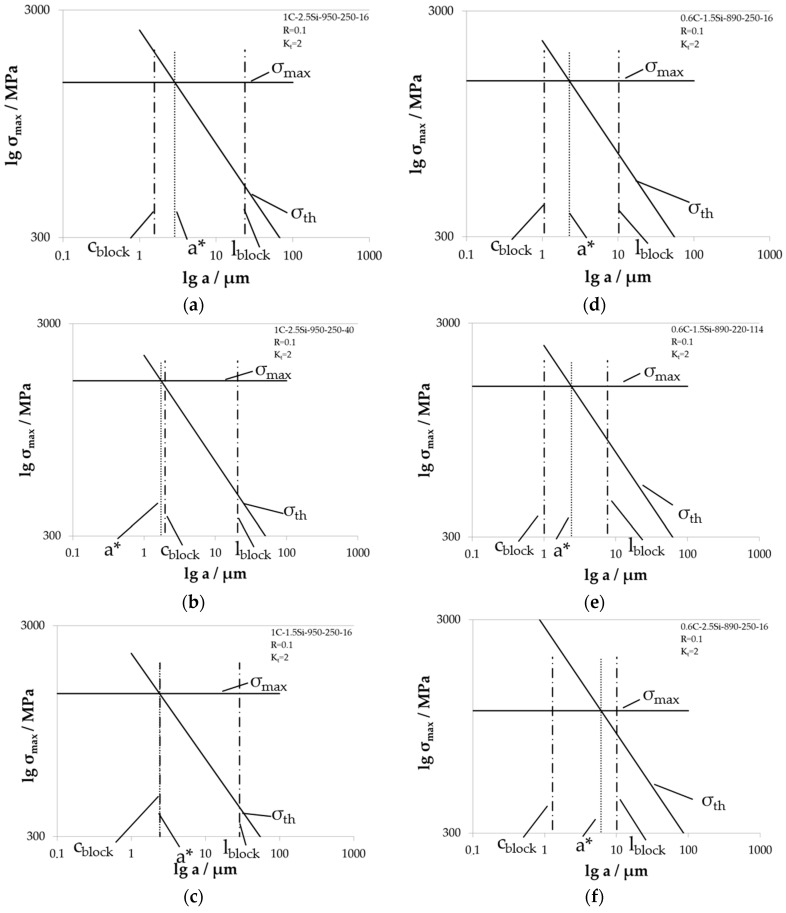
Individual Kitagawa diagrams for material states tested with load ratio *R* = 0.1 at specimens with notch factor *K_t_* = 2 for (**a**) 1C-2.5Si-950-250-16; (**b**) 1C-2.5Si-950-250-40; (**c**) 1C-1.5Si-950-250-16; (**d**) 0.6C-1.5Si-890-250-16; (**e**) 0.6C-1.5Si-890-220-114; and (**f**) 0.6C-2.5Si-890-250-16.

In Figure 14, individual Kitagawa diagrams built as described before for all materials tested with *R* = 0.1 at notched specimen with *K_t_* = 2 are shown. The two possible relations of critical crack length a* to bainite block width *c*_block_ can be distinguished in Figure 14. In the case of a larger critical crack length a* than the bainite block width *c*_block_, the pointed line which marks a* is on the right-hand side from the chain lain representing the bainite block width *c*_block_ (see Figure 14b,c). Additionally, the crystallographic bainite block length *l*_block_ is marked in the Kitagawa diagrams in Figure 14. For all variants, it is larger than the critical crack length a*. Obviously, the critical crack length a* lies in between the crystallographic bainite block width *c*_block_ and the block length *l*_block_. From this, it can be deduced that the bainite block boundaries act as barriers for a growing short fatigue crack because it grows inside the bainitic blocks until it reaches the critical crack length to change to long fatigue crack growth regime.

**Figure 15:**
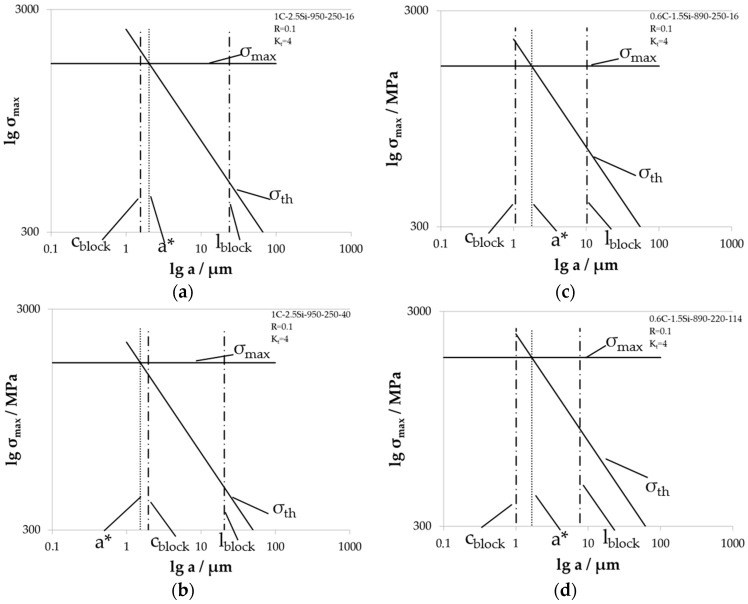
Individual Kitagawa diagrams for material states tested with load ratio *R* = 0.1 at notched specimens with *K_t_* = 4 for (**a**) 1C-2.5Si-950-250-16; (**b**) 1C-2.5Si-950-250-40; (**c**) 06C-1.5Si-890-250-16; and (**d**) 0.6C-1.5Si-890-220-114.

In Figure 15, the individual Kitagawa diagrams for microstructures tested at notched specimen with *K_t_* = 4 are shown. The determined critical crack length a* of these diagrams are also listed in Table 2. These values are smaller compared to the critical crack lengths determined at specimens with *K_t_* = 2 for the same microstructures. The sharper notch causes a higher local stress in the notch ground. With this higher local stress, the material is more critical for fatigue crack growth and a smaller critical crack length a* leads to the beginning of the long fatigue crack growth regime compared to a smoother notch. As for notched specimens with *K_t_* = 2, the size relationship of the critical crack length a* and the crystallographic bainite block width *c*_block_ is the same here. In the case of 1C-2.5Si-950-250-40, the crystallographic bainite block width *c*_block_ is larger than the critical crack length a*. In the other cases, the crystallographic bainite block width is smaller, i.e., the bainite blocks can be a barrier for a growing fatigue crack. The comparison of the critical crack length a* and the bainite block length *l*_block_ shows here as well that the bainite blocks are longer than the critical crack length a*. Again, the critical crack length a* lies in between the two dimensions of the bainite blocks, which obviously act as barriers for growing short cracks that are growing inside these blocks.

**Figure 16:**
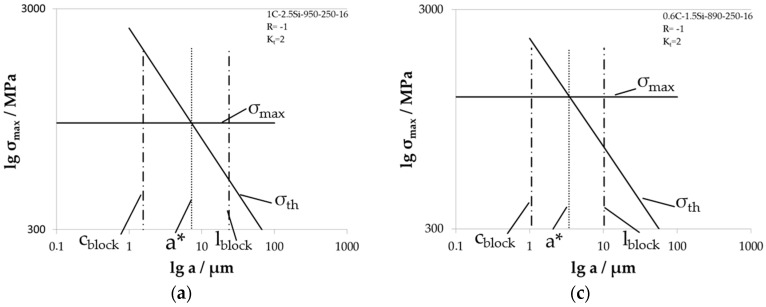

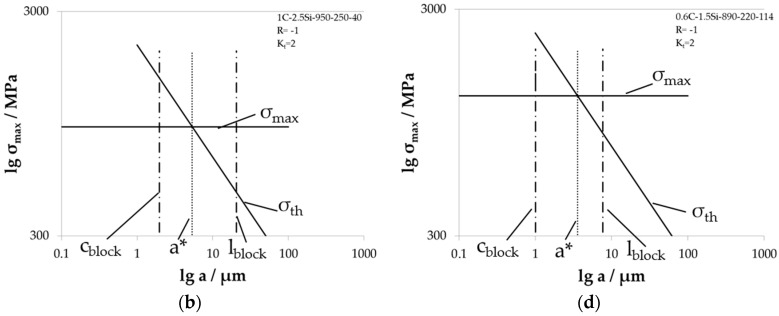
Individual Kitagawa diagrams for material states tested with load ration *R* = −1 at notched specimens with *K_t_* = 2 for (**a**) 1C-2.5Si-950-250-16; (**b**) 1C-2.5Si-950-250-40; (**c**) 06C-1.5Si-890-250-16; and (**d**) 0.6C-1.5Si-890-220-114.

In Figure 16, the individual Kitagawa diagrams for notched specimen with *K_t_* = 2 tested with load ratio *R* = −1 are shown. Here, it must be taken into account that the threshold value for long fatigue crack growth *K*_th_ was determined for a load ratio of *R* = 0.1. This could lead to small failure in the calculation. The determined values of the critical crack length a* are listed in Table 2. These sizes are all larger than the corresponding crystallographic bainite block width *c*_block_ due to the lower maximum stress in the notch ground. In addition, the critical crack length is smaller than the crystallographic bainite block length, i.e., in all of these cases, the bainite blocks plays a role for short fatigue crack growth, and cracks that are growing inside the bainite blocks can be decelerated until they reach the critical crack length a*.

Short fatigue cracks are highly influenced by barriers in the microstructure at which these cracks can be stopped, decelerated or deflected. Results from Kitagawa diagrams show that reaching the critical crack length and entering the long crack regime can be delayed by controlling the size relation between crystallographic bainite block width *c*_block_, bainite block length *l*_block_ and critical crack length a* in nanostructured bainitic steels. These diagrams show as well that a short fatigue crack grows inside the bainite blocks until it reaches the critical crack length a*. To have an influence on a growing fatigue crack, we need small bainite blocks compared to the critical crack length a* at which long fatigue crack growth starts. Then, a barrier for short fatigue crack growth exists at the boundaries of the crystallographic bainite blocks, and a short fatigue crack can be stopped or decelerated.

## 5. Conclusions

In this study, the fatigue and crack growth behavior of nanostructured bainitic steels was investigated. Together with a detailed microstructural analysis, several conclusions could be deduced from the gathered results:

- Features like bainite block sizes in nanostructured bainite can be quantitatively measured by light optical microscope with the same precision as by EBSD
- Nanobainitic specimens show fatigue failure from nonmetallic inclusions like often observed for high strength steels
- The size of the critical crack length a* is between the bainite block width *c*_block_ and the bainite block length *l*_block_
- The microstructural features governing the fatigue limit is the bainite block size, which is the main obstacle for short fatigue crack growth.

## Figures and Tables

**Table 1:** Heat treatment of the investigated specimens. *T_γ_* and *T_b_* are the austenitization and austempering temperature, respectively, and *t_b_* is the austempering time. The austenitization time is 1 h for all specimens.

Specimen	Casts	*T_γ_*, °C	*T_b_*, °C	*t_b_*, h
1C-2.5Si-950-250-16	industrial	950	250	16
1C-2.5Si-950-250-40	industrial	950	250	40
1C-1.5Si-950-250-16	laboratory	950	250	16
0.6C-1.5Si-890-250-16	industrial	890	250	16
0.6C-1.5Si-890-220-114	industrial	890	220	114
0.6C-2.5Si-890-250-16	laboratory	890	250	16

**Table 2:** Overview of the results of the microstructural analysis, *l*_block_ and *c*_block_, fatigue limits *σ*_a,50%_ for the different tested load ratios and notch geometries of the specimens, threshold values for long fatigue crack growth *K*_th_ determined by crack growth experiments and critical crack length a * determined for the different tested load ratios and notch geometries of the specimens determined out of Kitagawa diagrams; *) determined from specimens with notch factor *K_t_* = 1.89.

Material/Heat Treatment	*l*_block_	*c*_block_	*σ*_a,50%_			*K*_th_	a *		
			*K_t_* = 4	*K_t_* = 2	*K_t_* = 2	*K_t_* = 2	*K_t_* = 4	*K_t_* = 2	*K_t_* = 2
			*R* = 0.1	*R* = −1	*R* = 0.1	*R* = 0.1	*R* = 0.1	*R* = −1	*R* = 0.1
	µm	µm	MPa			MPam^1/2^	µm		
1C-2.5Si-950-250-16	23.7 ± 3.4	1.6 ± 0.2	210	445	310	5.3	2	7.2	2.9
1C-2.5Si-950-250-40	20.6 ± 4.5	2 ± 0.4	200	450 *)	335 *)	4.6	1.5	5.4	1.7
1C-1.5Si-950-250-16	22.7 ± 8.2	2.4 ± 0.4	-	-	310	4.8	-	-	2.4
0.6C-1.5Si-890-250-16	10.2 ± 1.7	1.1 ± 0.3	240	605	365	4.8	1.8	3.4	2.3
0.6C-1.5Si-890-220-114	7.7 ± 1.5	1 ± 0.2	230	605	350	5.1	1.7	3.6	2.4
0.6C-2.5Si-890-250-16	10.1 ± 2.6	1.3 ± 0.3	-	-	255	6	-	-	6.1

## Abbreviations

The following abbreviations are used in this manuscript:

SIF	Stress intensity factor
SEM	Second electron microcopy
XRD	X-ray diffraction
EBSD	Electron backscatter diffraction
LOM	Light optical microscopy

## References

[1] Caballero F.G., Bhadeshia H.K.D.H. (2004). Very strong bainite. Curr. Opin. Solid State Mater. Sci..

[2] Sourmail T., Smanio V., Ziegler C., Heuer V., Kuntz M., Caballero F.G., Garcia-Mateo C., Cornide J., Elvira R., Leiro A. (2013). Novel Nanostructured Bainitic Steel Grades to Answer the Need for High-Performance Steel Components (Nanobain).

[3] Sourmail T., Caballero F.G., Garcia-Mateo C., Smanio V., Ziegler C., Kuntz M., Elvira R., Leiro A., Vuorinen E., Teeri T. (2013). Evaluation of potential of high Si high C steel nanostructured bainite for wear and fatigue applications. Mater. Sci. Technol..

[4] Bhadeshia H.K.D.H. (2013). The first bulk nanostructured metal. Sci. Technol. Adv. Mater..

[5] Das Bakshi S., Shipway P.H., Bhadeshia H.K.D.H. (2013). Three-body abrasive wear of fine pearlite, nanostructured bainite and martensite. Wear.

[6] Solano-Alvarez W., Pickering E.J., Bhadeshia H.K.D.H. (2014). Degradation of nanostructured bainitic steel under rolling contact fatigue. Mater. Sci. Eng. A.

[7] Yang J., Wang T.S., Zhang B., Zhang F.C. (2012). High-cycle bending fatigue behaviour of nanostructured bainitic steel. Scr. Mater..

[8] Wang T.S., Yang J., Shang C.J., Li X.Y., Lv B., Zhang M., Zhang F.C. (2008). Sliding friction surface microstructure and wear resistance of 9SiCr steel with low-temperature austempering treatment. Surf. Coat. Technol..

[9] Zhang P., Zhang F.C., Yan Z.G., Wang T.S., Qian L.H. (2011). Wear property of low-temperature bainite in the surface layer of a carburized low carbon steel. Wear.

[10] Miller K.J. (1993). The two thresholds of fatigue behaviour. Fatigue Fract. Eng. Mater. Struct..

[11] Pham A.H., Ohba T., Morito S., Hayashi T. (2013). Energy stability of boundary between variants in lath martensite. J. Alloys Compd..

[12] Beladi H., Adachi Y., Timokhina I., Hodgson P.D. (2009). Crystallographic analysis of nanobainitic steels. Scr. Mater..

[13] Toji Y., Matsuda H., Raabe D. (2016). Effect of Si on the acceleration of bainite transformation by pre-existing martensite. Acta Mater..

[14] Rementeria R., Morales-Rivas L., Kuntz M., Garcia-Mateo C., Kerscher E., Sourmail T., Caballero F.G. (2015). On the role of microstructure in governing the fatigue behaviour of nanostructured bainitic steels. Mater. Sci. Eng. A.

[15] Samuels L.E. (1999). Light Microscopy of Carbon Steels.

[16] (2011). ASTM E647-11: Standard Test Method for Measurement of Fatigue Crack Growth Rates.

[17] Erdogan F., Ratwani M. (1970). Fatigue and fracture of cylindrical shells containing a circumferential crack. Int. J. Fract. Mech..

[18] Nolze G. (2004). Characterization of the fcc/bcc orientation relationship by EBSD using pole figures and variants. Z. Metall..

[19] Sourmail T., Galtier A., Sanz R.P., Jannisch R., Sampath S., Müller I., Kerscher E., Rementeria R., Garcia-Mateo C., Caballero F.G. (2016). Understanding the basic mechnaism to optimize and predict in service properties of nanobanitic steels. Res. Fund Coal Steel.

[20] Murakami Y. (2012). Materials defects as the basis of fatigue design. Int. J. Fatigue.

[21] Murakami Y. (2002). Metal Fatigue: Effects of Small Defects and Nonmetallic Inclusions.

[22] Radaj D. (2014). State-of-the-art review on extended stress intensity factor concepts. Fatigue Fract. Eng. Mater. Struct..

[23] Grad P., Reuscher B., Brodyanski A., Kopnarski M., Kerscher E. (2012). Analysis of the crack initiation at non-metallic inclusions in high strength steels. Pract. Metallogr..

[24] Grad P., Reuscher B., Brodyanski A., Kopnarski M., Kerscher E. (2012). Mechanism of the fatigue crack initiation and propagation in the very high cycle fatigue regime of high-strength steels. Scr. Mater..

[25] Morales-Rivas L. (2016). Microstructure and Mechanical Response of Nanostructured Bainitic Steels.

